# Probabilistic early warning signals

**DOI:** 10.1002/ece3.8123

**Published:** 2021-09-26

**Authors:** Ville Laitinen, Vasilis Dakos, Leo Lahti

**Affiliations:** ^1^ Department of Computing University of Turku Turku Finland; ^2^ Institut des Sciences de l’Evolution de Montpellier (ISEM) University of Montpellier Montpellier France

**Keywords:** early warning signals, probabilistic programming

## Abstract

1. Ecological communities and other complex systems can undergo abrupt and long‐lasting reorganization, a regime shift, when deterministic or stochastic factors bring them to the vicinity of a tipping point between alternative states. Such changes can be large and often arise unexpectedly. However, theoretical and experimental analyses have shown that changes in correlation structure, variance, and other standard indicators of biomass, abundance, or other descriptive variables are often observed prior to a state shift, providing early warnings of an anticipated transition. Natural systems manifest unknown mixtures of ecological and environmental processes, hampered by noise and limited observations. As data quality often cannot be improved, it is important to choose the best modeling tools available for the analysis.
2. We investigate three autoregressive models and analyze their theoretical differences and practical performance. We formulate a novel probabilistic method for early warning signal detection and demonstrate performance improvements compared to nonprobabilistic alternatives based on simulation and publicly available experimental time series.
3. The probabilistic formulation provides a novel approach to early warning signal detection and analysis, with enhanced robustness and treatment of uncertainties. In real experimental time series, the new probabilistic method produces results that are consistent with previously reported findings.
4. Robustness to uncertainties is instrumental in the common scenario where mechanistic understanding of the complex system dynamics is not available. The probabilistic approach provides a new family of robust methods for early warning signal detection that can be naturally extended to incorporate variable modeling assumptions and prior knowledge.

## Graphical abstract

The article is concerned with early warning signals in one‐dimensional dynamical systems. We present a new probabilistic method for detecting rising lag‐1 autocorrelation and benchmark it against previously published approaches.

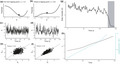

## 1 INTRODUCTION

Ecosystems often respond to environmental drivers in a gradual fashion; small changes in external conditions lead to small changes in the system (Scheffer et al., 2001). However, even stable and resilient systems may unnoticeably drift toward a critical threshold—a tipping point where an abrupt shift to an alternative stable state may occur (Arani et al., 2021; Lenton, 2020; Scheffer et al., 2001, 2009). Whereas such sudden changes have been reported for instance in the context of deforestation (Scheffer et al., 2001), loss of vegetation in shallow lakes (Scheffer & Nes, 2007), in human gut microbiome in connection to western lifestyle (O’Keefe et al., 2015), and collapsing fish populations (Pedersen et al., 2017), critical transitions are notoriously difficult to predict. As the anthropogenic impact is potentially driving ecosystems, the climate, and our bodies closer to such tipping points with possibly irreversible consequences, our ability to anticipate critical transitions in complex natural and social systems has become perhaps more important than ever.

Despite the difficulties in predicting such critical transitions (Clements & Ozgul, 2018; Hastings & Wysham, 2010), generic early warning signals (EWS) have been identified and reported in both theoretical and real dynamical systems in various different fields (Dakos, Carpenter, et al., 2012; Scheffer et al., 2009). In ecology, EWS have been shown to precede population collapses and ecosystem state shifts (Carpenter et al., 2011; Drake & Griffen, 2010; Wang et al., 2012). These warning signals are statistical indicators that typically target critical slowing (CSD), a symptom of a system close to a tipping point (van Nes & Scheffer, 2007), in biomass, population size, or some other measurable aspect of the system. CSD refers to reduced ability to recover from perturbations, and it has been measured with various statistics that quantify the system state and its observed changes over time.

Broadly speaking, EWS detection methods have been categorized into model‐based and metric‐based indicators (Dakos, Carpenter, et al., 2012). Metric‐based methods measure the dynamical properties of a time series without using an explicit model. Decreasing recovery rate, or increasing of its reciprocal lag‐1 autocorrelation (Ives, 1995), is one of the most utilized ones and is computed simply as the correlation between consecutive observations of the variable of interest. Other metric‐based indicators include increasing variance, skewness, kurtosis, and changes in spectral properties (Dakos, Carpenter, et al., 2012).

Model‐based indicators look for EWS by fitting some model to the data and investigating the output. Lag‐1 autocorrelation, for instance, can be estimated with autoregressive AR(p) processes, as one of the model parameters directly measures it. Extendability of models is the key advantage with the model‐based approach and several variants of the standard AR(1) process have been studied in EWS context. With lags p>1, it is possible to model longer term memory effects which may also improve the model fit if the data are non‐Markovian (Ives & Dakos, 2012). The time‐varying AR(*p*) process is a further generalization that allows time‐varying coefficients and has been shown to provide enhanced performance in certain applications (Ives & Dakos, 2012). In this work, we provide a new probabilistic formulation of the time‐varying AR(1) process and compare its performance against two previously suggested autoregressive models.

**FIGURE 1:**
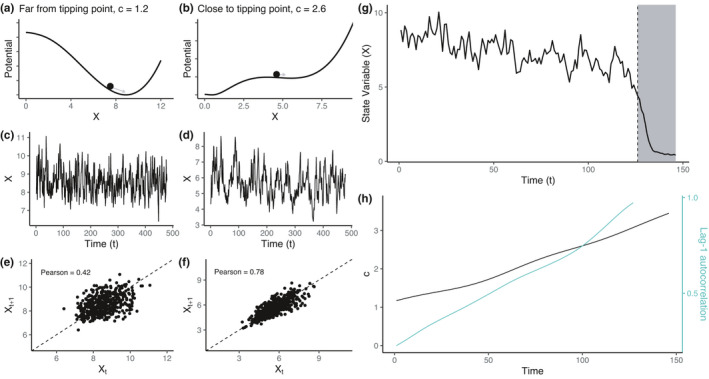
Illustration of increasing lag‐1 autocorrelation prior to a state shift in a complex dynamical system. (a) When the dynamical system is far from a tipping point, the underlying potential landscape has a relatively clear minimum which strongly attracts the system state, represented by the black ball. (b) Close to the tipping point, the potential minimum has become shallow and the attraction is weaker. (c) Time series simulated far from the tipping point resembles white noise whereas closer to it the dynamics have slowed down (d). (e, f) System state mapped against successive time points shows changes in lag‐1 autocorrelation. (g) Time series simulated from an ecological model where a state shift can be observed at the dashed vertical line. (h) The model parameter c (black) is a bifurcation parameter that drives the system toward a tipping point when increased. Lag‐1 autocorrelation estimated from the example time series (green) increases prior to the state shift and signals a heightened risk for transitioning

Despite the wealth of available indicators, it is understood that EWS detection is challenging, especially in real data. A sufficiently large sample size and observation density at relevant time scales are needed to recover a robust signal (Arkilanian et al., 2020; Clements et al., 2015; Dakos, Carpenter, et al., 2012). Often real data do not match these criteria and can include some additional complicating factors, such as high and nonstationary levels of noise, nonuniform observations, and unknown changes in data‐generating mechanisms. Moreover, while the perspective of critical slowing down due to changing potential wells (see Figure 1) is theoretically appealing and guiding intuition, it is has been reported that variable behaviors of real systems can be observed at the vicinity of a tipping point. Thus, real systems may display some, but not necessarily all, EWS. Furthermore, there is no one‐size‐fits‐all model, or even way of applying a chosen model, as certain modeling choices may have large effects on the qualitative nature of the results (Dakos, Carpenter, et al., 2012). For these reasons, it is apparent that any EWS analysis of a real system should be interpreted cautiously.

To the best of our knowledge, all EWS methods published thus far have been developed within the frequentist framework. Probabilistic Bayesian frameworks for EWS detection and analysis could therefore complement the existing literature and potentially offer some benefits over previously proposed techniques for instance in parameter inference, uncertainty quantification, and incorporation of prior knowledge (Gelman et al., 2013). For instance, in the frequentist framework, the degree of statistical certainty of EWS is often quantified with surrogate data analysis methods. These require specification of a null model for the data, which unnecessarily increases the complexity and uncertainties in the EWS detection scheme. As we show here, uncertainty quantification is naturally built into the probabilistic equivalent. Furthermore, in applied settings, there is often at least some prior information available about the system under investigation. The probabilistic framework provides interesting opportunities for incorporating such prior information in the models.

## 2 METHODS

This work introduces a probabilistic approach to early warning signal detection. We will first show how the standard autoregressive process has been used in early warning signal detection, then describe its time‐varying extension, and finally present a probabilistic formulation of such time‐varying model. We will then discuss the specific aspects and shortcomings of each model and show that the probabilistic formulation exhibits some attractive properties compared to the alternatives. We use simulation experiments to benchmark the new approach and also display its performance on experimental data from real case studies.

### 2.1 Standard autoregressive AR(1) process as an early warning signal

The autoregressive AR(1) process has been reported to have good overall performance as an early warning signal (Dakos et al., 2008, 2012b). Let us first provide an overview of this process and its application as an early warning signal (Dakos, Carpenter, et al., 2012; Scheffer et al., 2009). The AR(1) process is defined with the recursion.

(1) $$X_{t+1}=\mu +\phi \left(X_{t}‐\mu \right)+\epsilon_{t}$$

where $X_{t}$ is the state variable at time t, μ the process mean, ϕ the autoregressive coefficient, and $\epsilon_{t}$ a Gaussian random variable with mean zero and standard deviation σ. The process can be used to model stochastic dynamics of a property, such as biomass or abundance, around a single stable state μ with a mean‐reversion tendency, or *drift*, whose strength and characteristics are determined by ϕ. When ϕ is close to zero any stochastic events and perturbations are suppressed rapidly, whereas values approaching one result in a weaker pull toward the attractor μ. If ϕ=1, the process becomes Brownian motion, and if ϕ>1, the mean level μ becomes a repeller. Observing an increasing ϕ suggests that the mean‐reverting tendency is decreasing and provides a quantitative method to observe slowing dynamics. This critical slowing down has been reported to precede catastrophic transitions and is a theoretically justified EWS (Scheffer et al., 2009).

A practical shortcoming in fitting the AR(1) process to the observed data is that it yields only a single value for ϕ, whereas changes in this term over time need to be observed in order to use it as an EWS. The standard solution is to use the sliding window approach that divides the time series into overlapping segments (Dakos, Carpenter, et al., 2012). The AR(1) process is then fit to each of these windows, providing a trajectory ϕ that can be used to assess changes in the system dynamics. This approach treats the windows separately, meaning that any potential continuity or momentum of parameters is not modeled. Moreover, the estimated trajectory reflects average changes in the windows and cannot be used to pinpoint exact locations in the data where potential sudden dynamical changes occurred.

As a preprocessing step, the data are often detrended and the model fitted to the residuals, which helps to avoid spurious effects of nonstationary trends unrelated to the intrinsic stability of the system (Dakos, Carpenter, et al., 2012). We used Gaussian kernel smoothing (utilizing R function *stats::ksmooth*) to detrend the raw time series data. We then fitted the AR(1) process to the residuals with ordinary least squares optimization (*stats::ar.ols* function).

There is no generally accepted way to set the kernel smoothing bandwidth or sliding window length in the early warning context. Here, except where otherwise indicated, we set the sliding window length to 50%, a figure often encountered in EWS literature, and chose the time series smoothing bandwidth bw with "Scott's rule":

$$\text{bw}=1.06\cdot \text{min}\left(\hat{\sigma },\frac{\text{IQR}}{1.34}\right)\cdot n^{‐1/5},$$

where $\hat{\sigma }$ is the standard deviation, IQR the interquartile range of data, and n the sample size.

Following typical practices in the EWS literature, we quantify the trend in the estimated trajectory of ϕ with Kendall's rank correlation, τ, between ϕ and time t (Dakos, Carpenter, et al., 2012; Scheffer et al., 2009). This test statistic is defined in terms of rank orders: $\tau =N_{\text{concordantpairs}}‐N_{\text{disconcordantpairs}}/N_{\text{allpairs}}$, where N refers to the number of elements in the set denoted by the subscript. A pair of observations $\left(t_{i},\phi_{i}\right),\left(t_{j},\phi_{j}\right)$ is said to be concordant if $t_{i}<t_{j}$ implies $\phi_{i}<\phi_{j}$ and disconcordant otherwise.

We estimated the statistical significance of a positive trend (τ>0) utilizing surrogate data analysis, following an approach used in Dakos, Carpenter, et al. (2012). The approach is to simulate N time series from an ARMA(p, q) model that best fits the original data (in terms of the Akaike information criterion; p,q = 1,…,5), estimate ϕ in each of these simulations and then compute the corresponding τ’s. This results in an approximate sampling distribution T for the test statistic τ that enables performing hypothesis testing. We used the one‐tailed test, meaning that we considered the trend significant at the level α, when the original estimate is at the 1‐α upper percentile of the sampling distribution T. We set the alpha level to α=0.1. Although this is somewhat higher than the typically used levels for statistical significance, we consider this useful in the EWS context where observing a warning of a potentially impending catastrophe may be more important than having a practical statistical certainty.

### 2.2 Nonprobabilistic time‐varying autoregressive process

A more agile variant of the standard AR(1) process can be formulated by letting the mean and autoregressive parameters μ and ϕ in Eq. 1 vary in time. The time‐varying AR(1) is denoted as TVAR(1) and defined as the recursion (Ives & Dakos, 2012).

(2) $$X_{t+1}=\mu_{t}+\phi_{t}\left(X_{t}‐\mu_{t}\right)+\epsilon_{t}.$$

The TVAR(1) model is fitted once across the entire time series and removes the need to use sliding windows. This simplifies the analysis, as the conclusions are independent of the sliding window length selection. Moreover, there is no need to detrend the data before inference inference, as the time‐varying mean parameter can learn any nonstationary mean level variations.

The model needs to be regularized, however, as the degrees of freedom is too large for the model to be identifiable. We achieve this by utilizing time‐varying ordinary least squares kernel regression (with the Nadaraya–Watson estimator) as implemented in the *tvReg::tvAR* function (Casas & Fernandez‐Casal, 2019). The kernel and its bandwidth control the level of smoothing by adjusting the weight that the neighboring time points have on estimates at t. We used the Gaussian kernel that is of the form $K\left(x\right)=\left(1/2\pi \right)\exp\left(‐x^{2}/2\right)$, and unless otherwise noted, we chose the bandwidth value with leave‐one‐out cross validation.

We determined the level of statistical significance of the parameter trends with surrogate data methods.

### 2.3 The probabilistic formulation

Now, we propose a probabilistic variant of the TVAR(1) process, denoted pTVAR(1), as an alternative method for detecting early warning signals.

The motivations for investigating the probabilistic alternative include enhanced capabilities to regularize and extend the models and to include prior assumptions of the system (Gelman et al., 2013). A notable practical advantage is that the probabilistic model readily allows the estimation of statistical significance in the early warning indicator trend without resorting to surrogate data analysis methods that add an unnecessary level of complexity on the analysis.

The probabilistic formulation is based on the time‐varying autoregressive process of order 1 in Eq. 2. The likelihood of the TVAR(1) parameters, given the observations $X_{t}$ is obtained as follows:

$$L\left(\mu_{t},\phi_{t},\sigma |X_{t}\right)=\prod_{t=1}^{N‐1}N\left(X_{t+1}|\mu_{t}+\phi_{t}\left(X_{t}‐\mu_{t}\right),\sigma^{2}\right),$$

where the notation $N\left(\mu ,\sigma^{2}\right)$ refers to the normal distribution with mean μ and variance $\sigma^{2}$.

To complement the likelihood functions, we add Gaussian process (GP) priors for the trajectories of $\phi_{t}$ and $\mu_{t}$. A Gaussian process $GP\left(M,\Sigma \right)$ is a collection of random variables, any finite subset of which is multivariate normally distributed with mean M and covariance Σ and provides a means for nonparametric regression (Rasmussen & Williams, 2006). We use the Matèrn‐3/2 covariance function that defines the covariance between two random variables $X_{i}$ and $X_{j}$ as $k_{3/2}\left(\rho ,\alpha \right)=\alpha^{2}\left(1+\left(\sqrt{3}r/l\right)\right)\exp\left(‐\left(\sqrt{3}r/l\right)\right),$ where $\alpha^{2}$ is the process variance, ρ the length scale and $r=\left|X_{i}‐X_{j}\right|$ (Rasmussen & Williams, 2006; Stein, 1999). This choice constrains the parameter trajectories over time to be continuous and differentiable, providing a natural assumption that we impose to regularize the model fit (Scheffer et al., 2009). The length scale controls the range at which inference for different time points are affected and the overall dependency between consecutive time points and, in effect, smoothens the posterior trajectories. The parameter $\alpha^{2}$ determines the average distance from the mean parameter M.

In general, Matèrn covariance functions form a larger class of covariance structures characterized by a parameter ν that controls the smoothness of the sample trajectories. Here, we use ν=3/2 which results in once differentiable trajectories. The rationale behind the choice is that ν=1/2 produces unnecessarily rough functions, whereas values of ν>5/2 would be unrealistically smooth, see Rasmussen and Williams (2006) and Stein (1999) for details. We also experimented with ν=5/2 and recovered similar results as with ν=3/2.

The full generative probabilistic model is.

$$\begin{array}{c} \phi_{t}∼\text{GP}\left(M_{\phi },k_{3/2}\left(\rho_{\phi },\alpha_{\phi }\right)\right)\\ \mu_{t}∼\text{GP}\left(M_{\mu },k_{3/2}\left(\rho_{\mu },\alpha_{\mu }\right)\right)\\ \sigma ∼\text{half}-N\left(0,1\right)\\ \epsilon_{t}∼N\left(0,\sigma^{2}\right)\\ X_{t+1}=\mu_{t}+\phi_{t}\left(X_{t}-\mu_{t}\right)+\epsilon_{t} \end{array}$$

Before fitting, we standardized the state variable X by subtracting mean $\bar{X}$ and dividing by standard deviation $SD\left(X\right)$. Standardization allows us to automatically set the hyperparameter values for $\mu_{t}$ to $M_{\mu }=0$ and $\alpha_{\mu }=1$, respectively. We chose the same values for the autoregressive parameter's hyperparameters: $M_{\phi }=0$ and $\alpha_{\phi }=1$. Unless otherwise denotes, we set length scale parameters $\rho_{\mu }$ and $\rho_{\phi }$ to the length of time series. This choice avoids overfitting and highlights the long‐term trends in data, which is of the greatest interest in EWS context.

**FIGURE 2:**
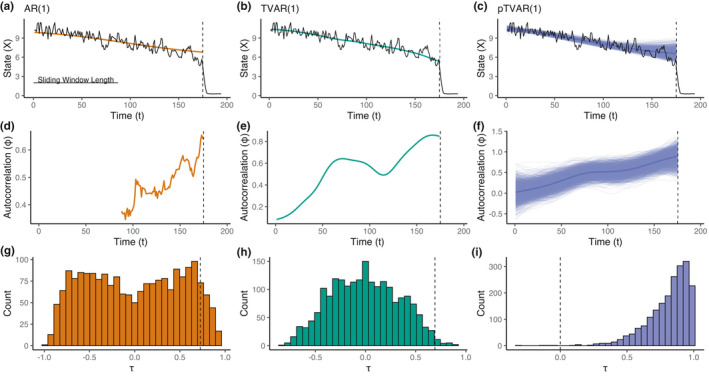
Early warning signal detection with simulated data. (a, b, c) The observed system state X as a function of time (black curve). The system gradually approaches a tipping point before a state transition ultimately occurs around the dashed vertical line (*T* = 175). The colored lines correspond to the estimated time series trend based on the Gaussian kernel smoother applied on the data, the estimated TVAR(1), and the posterior samples of the pTVAR(1) mean parameter. Length of the sliding window was set to 50% of time series length prior to the transition. (d, e, f) Increasing autocorrelation can be observed prior to the state shift with all methods. The figure shows ordinary least squares estimates for autoregressive parameter for the standard (brown) and time‐varying (green) autoregressive models, and the posterior samples and mean for the probabilistic method (purple). (g, h) approximate sampling distributions for the autoregressive parameter trends, $\tau_{\phi }$, for AR(1) and TVAR(1) are obtained with surrogate data analysis. Kendall's $\tau_{\phi }$ quantifies the association strength between the early warning indicator $\phi_{t}$ and time. A significant positive correlation indicates increasing risk of a state shift. The dashed vertical line denotes the point estimate and the proportion of the sampling distribution above it provides the *p*‐values .095 and .013 for the AR(1) and TVAR(1), respectively. (i) Posterior distribution of $\tau_{\phi }$ obtained with pTVAR(1). Altogether 99.95% of the posterior samples are positive, providing strong evidence for an increasing trend that is interpreted as an EWS. The probabilistic *p* value is the proportion of the τ posterior that is not positive. Note that the posterior is not a sampling distribution, which explains the qualitatively different shape of the distribution, compared to g and h

A convenient property of our probabilistic method is that each posterior point corresponds to a specific parameterization $\phi_{t}$, $\mu_{t}$ (see Figure 2). The benefits are twofold. First, as $\mu_{t}$ represents the time‐varying mean levels of the data, the uncertainty in the posterior of $\mu_{t}$ softens the choice we make when setting $\rho_{\mu }$. Thus, each posterior sample can be interpreted as a specific, semi‐automatically determined smoothing function. Second, posterior variation of $\phi_{t}$ can be used to directly produce a posterior distribution of the Kendall τ, by computing τ for each posterior sample of $\phi_{t}$. Analogously to the nonprobabilistic case, we required $100\cdot \left(1‐\alpha \right)\text{\textbackslash{}\%}\;$ of the $\tau_{\phi }$ posterior evidence for τ>0 with detection level α=0.1.

See Table 1 for comparison of autoregressive model properties and Figure 2 for an illustration of the fitting procedure.

**TABLE 1:** Comparison of autoregressive‐1 model properties

Model	Probabilistic	Sliding window	Smoothing	Statistical significance
AR(1)	No	Yes	Separate	Surrogate analysis
TVAR(1)	No	No	In model	Surrogate analysis
PTVAR(1)	Yes	No	In model	In model

We used the probabilistic programming language Stan (Stan Development Team, 2020) to fit the pTVAR(1) process. Detailed fitting information is provided in the Appendix 1 and the source code is available in the online repository https://zenodo.org/record/4638525.

### 2.4 Simulation model

We used simulated data to benchmark the alternative methods. We simulated data with state shifts using a well‐studied ecological model that exhibits alternative stable states (May, 1977). The model consists of logistic growth limited by a carrying capacity, a harvest term that models removal of biomass, and state‐dependent stochastic variation levels. The model is defined by the stochastic differential equation.

$$\begin{array}{c} dX=\left(rX\left(1‐\frac{X}{K}\right)‐c\frac{X^{2}}{X^{2}+h^{2}}\right)dt+\sigma XdW_{t}, \end{array}$$

where X is the state variable, r is the growth rate, K the carrying capacity, c the harvest rate, h the half‐saturation constant, σ the diffusion coefficient, and $dW_{t}$ the Wiener process.

In order to ensure robustness of results to variations in model parameters, including time series length T, we generated 250 time series with randomly sampled parameters at each replication using the following distributions: $T∼\text{Unif}\left(50,200\right),r∼N\left(1,0.1\right),K∼N\left(10,1\right),h∼N\left(1,0.1\right),\sigma ∼N\left(0.1,0.01\right)$ and $c∼\text{GP}\left(\delta_{c},k_{\mathrm{SE}}\left(\rho_{c},\alpha_{c}\right)\right)$, where $\alpha_{c}∼\text{Unif}\left(0.05,0.25\right)$, $\rho_{c}∼\text{Unif}\left(10,50\right)$, and $k_{\mathrm{SE}}$ is the squared exponential kernel (Rasmussen & Williams, 2006). The sampling distributions are based on previous studies (Dakos, Carpenter, et al., 2012).

The system's equilibrium structure can be controlled with the harvest parameter c. For example, with r=1,K=10 and h=1, the critical points are located at $c_{1}\approx$ 1.791 and $c_{2}\approx$ 2.604 and a bistable regime exists between these values. We sampled c from a Gaussian process with the mean parameter $\delta_{c}$ linearly grown along the simulation time range from 1 to 3.5, well above the critical threshold value. This produces random trajectories of c that fluctuate around the linearly increasing trend. We chose the sampling distributions for the other parameters so that simulations with realistic signal‐to‐noise‐ratios and sample sizes were achieved.

In order to measure ability of the models to separate true warning signals from random variations generated by stationary dynamics, we also simulated data with approximately constant underlying conditions and no state shift. To this end, we removed the linear trend in $\delta_{c}$, set it to a constant value of one and simulated 250 replications with same parameters and sample sizes as in the data with state shifts. The time series lengths were matched with the shifting data prior to the shift.

In addition, we simulated data with varying levels of observation error. We simulated error processes from $N\left(0,\sigma^{2}\right)$ with values of σ of 0.2, 0.4, 0.6, 0.8, and 1. The error processes were added to the same realizations of state shifting and stationary data as above to achieve full comparability. We used the Euler–Maruyama discretization simulation scheme with step size 0.01 and random initial values $X_{t=0}∼N\left(K_{i},\sigma_{i}\right)$ for each replication i=1,…,250. We assessed locations of the change points visually and used only the part before the shift in the analysis. The average sample size was 93.4 with standard deviation 37.7 and range 17–189.

### 2.5 Model performance

We evaluated model performance based on true‐positive rate (sensitivity), true‐negative rate (specificity), and F1 score in simulated data (see *Simulation model*). We considered a positive trend (τ>0) at the selected α level as an EWS as described above and classified the simulated time series into two groups with and without observed EWS, respectively. Subsequently, we estimated the sensitivity, specificity, and overall accuracy in recovering EWS in simulated time series with a known state shift.

Moreover, we computed the mean squared error (MSE) between the autocorrelation estimated with models and a simulation approximation. We computed the approximation by simulating a realization of the generative model in equation 4 at each time point using the same parameters as in the original simulation. We then computed the sample lag‐1 autocorrelation for each of these realizations, thus generating an approximate "true" autocorrelation trajectory. This approximation method becomes unreliable in the bistable regime, as the simulations visit of both the alternative stable states and the computed autocorrelation reflects properties of both of these states. In order to get a realistic picture of the true autocorrelation, we smoothed the approximated trajectory with Gaussian kernel smoothing and then discarded the time points after the maximal value. We checked the robustness of results with two other error metrics, root mean squared error, and mean absolute error, and recovered qualitatively similar results.

### 2.6 Real data

In addition to the simulation benchmark, we analyzed three publicly available data sets with the presented autoregressive models.

The first data consist of 428 ocean sediment measurements of CaCo_3_ used as a proxy for the climate (Tripati et al., 2005). The measurements range a period of approximately 39 to 32 million years ago and contain a state shift which has been attributed to the end of the so‐called greenhouse Earth period. These data were analyzed from the EWS perspective in Dakos et al. (2008). The time points of these data are not precisely equidistant, but as reported in Dakos et al. (2008), the raw data give essentially similar results as the interpolated equidistant measurements.

The second data set consists of high‐frequency measurements of an experimental cyanobacteria community. The experimental setup comprised of a chemostat with cyanobacteria evolving under gradually increasing, increasingly detrimental levels of light over 29 days until population collapse. The microcosm was perturbed every 4–5 days by removing approximately 10% of the biomass via dilution. The sample size of the data was 7,784, but we subsampled this by using every 15th observation in the analysis leaving 454 time points for EWS analysis.

The third data consist of 188 stool samples of a single subject (Subject B in the original work) over 318 days. The taxonomic profile of these samples was assessed with 16S rRNA amplicon sequencing. We aggregated the data to genus level and used the centered logarithm (CLR) transform to take into account the compositionality bias. We visually identified genera that experienced a state shift at time point 155 and chose the genus as an *Akkermansia* example case. There were 127 time points before the state shift with the average time between observation 1.2 days, indicating that a large majority of the samples were taken one day apart.

We chose the hyperparameters so that the fitted trend did not overfit or underfit the data and that at the same time longer term trends in the autoregressive parameter were highlighted. We used length scale parameters $\rho_{\mu }=15,\rho_{\phi }=100$; $\rho_{\mu }=227,\;\rho_{\phi }=454;$ and $\rho_{\mu }=32,\;\rho_{\phi }=127$ for the climate, cyanobacteria, and gut microbiome time series, respectively.

In all cases, we only used the data prior to the shift in the EWS analysis.

## 3 RESULTS

This section provides a systematic benchmarking and comparison of the alternative methods presented in *Methods*.

### 3.1 Simulation experiments

#### 3.1.1 Bandwidth scan

First, we examined how EWS detection depends on the level of smoothing incorporated in the models. We tested this on two representative example time series of equal length, one with an induced state shift (and slowing dynamics) and one with constant conditions. We varied the length scale in the probabilistic method and the smoothing bandwidth in comparison models and checked the dependence of EWS metrics and general fit on these parameters. We used bandwidth and length scale values ranging from 0.05 to 1 in increments of 0.05 times the time series length. Although these parameters are not strictly corresponsive, they are analogous in that they are responsible for smoothing the estimated parameter trajectories.

**FIGURE 3:**
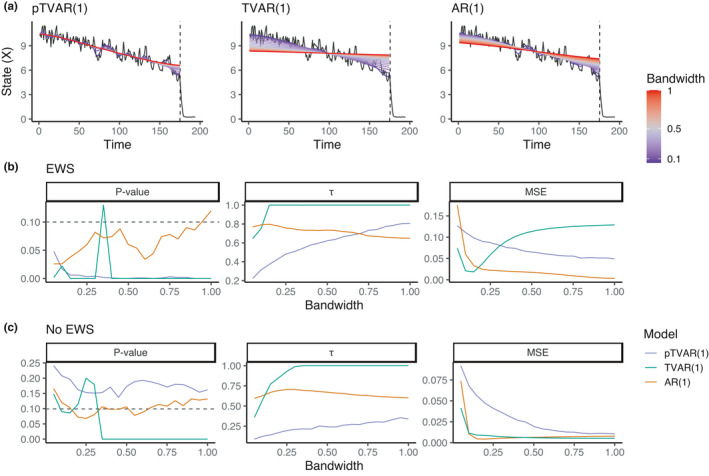
EWS detection accuracy depends on the smoothing bandwidth. (a) Fit for the observed trend for the probabilistic and comparison models in time series that include an EWS. The fit color indicates the bandwidth as a proportion of time series length, ranging from 0.05 to 1. Only the time points before the dashed vertical line were used in the inference. (b) EWS metrics as a function of bandwidth provide information of the expected true‐positive rate. *p*‐Values for the two TVAR(1)‐based methods exhibit similar significant ranges although the nonprobabilistic model exhibits some volatility. Increasing autocorrelation trend measured with τ is a standard EWS, here shown as a function of bandwidth. MSE is the mean squared error between the approximated autocorrelation trajectory and posterior mean autocorrelation (purple) or classical point estimates (green, brown) based on the simulation equation (see *Methods*). (c) EWS metrics for data without EWS provides information of the expected false‐positive rate

Whereas the comparison methods began to underfit at larger bandwidth values, the probabilistic model fitted accurately to the trend in the data for all used length scale values, see Figure 3. The model fit was assessed visually. The models produced similar and accurate fits on the data with constant conditions (data not shown).

All models were able detect EWS with statistical significance below the set α level. The TVAR(1) model *p*‐values displayed some inconsistent behavior in terms of a sudden spike at bandwidth value of .35. In the data without EWS, TVAR(1) produced false positives with larger bandwidth values. With pTVAR(1), *p*‐values remained consistently above .1, and the *p*‐value for AR(1) was close to .1 at all bandwidth levels.

Finally, we computed the mean squared error (MAE) between the autocorrelation estimates and approximated autocorrelation (see *Methods* section for details). In the EWS data, TVAR(1) had a minimum at bw = 0.15 but increased drastically at larger bandwidths, whereas for pTVAR(1), the error decreased as a function of length scale. For AR(1), the error was the lowest apart from the smallest values of bw. In the data without EWS, the comparison methods achieved low MSE values at practically all bandwidth values, compared to the probabilistic model.

#### 3.1.2 Multiple time series

As it is difficult to comprehensively assess and compare model performance in a single, relatively short time series, we next tested EWS detection accuracy in a large collection of such time series.

**FIGURE 4:**
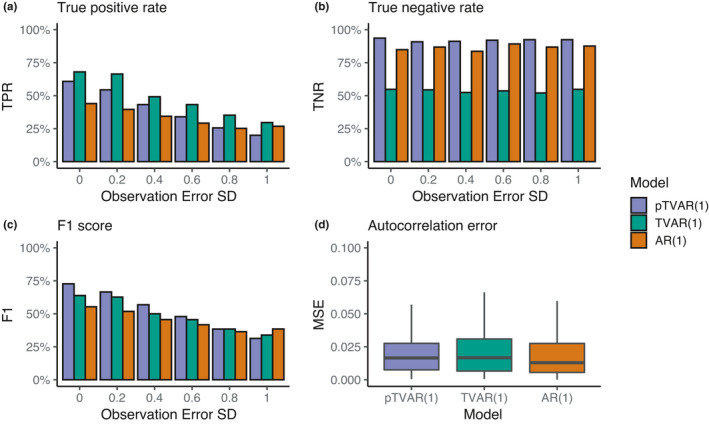
Early warning detection performance in simulated data (with 500 replications) across varying observation error levels. True‐positive rate (a), true‐negative rate (b), and F1 score (c) show the impact of observation error standard deviation on classification performance of early warning signals. (d) Mean squared error between the estimated and approximate autocorrelation trajectories. The MSE are based on data with and without EWS. Outliers have been omitted from the figure for clarity. We computed the approximated autocorrelations using the simulation equations (see *Methods*)

In terms of true‐positive rate, the proposed probabilistic model performed slightly worse than the comparison models (Figure 4). The AR(1) model performance was poorer compared to the time‐varying variants. The finding was consistent in data with higher observation error levels and, expectedly, performance of all models decreased as a function of the noise level.

In specificity, we found that performance of TVAR(1) was considerably worse than that of pTVAR(1) and the AR(1) models. This is in line with the results of the bandwidth scan as TVAR(1) seems to suffer from high amount of falsely detected EWS. Sensitivity seemed to be unaffected by observation error as true‐positive rates remained at the same level with all noise levels.

We also used the F1 score to get a view of the overall classification accuracy. Here, the probabilistic model achieved best performance at most levels of observation error, with the differences becoming smaller at higher error levels.

In mean squared error between the approximated and inferred autocorrelation trajectories, we found no meaningful differences between the different models.

### 3.2 Real scenarios

Next, in order to demonstrate the proposed method on real data, we re‐analyzed data sets from three previous studies where shifts between alternative stable states were observed. Two of these, analyses of a paleoclimatic time series (Dakos et al., 2008) and an experimental cyanobacteria population (Veraart et al., 2012), reported increasing autocorrelations before a state shift. Here, we replicate the findings of these studies and then give first preliminary evidence that critical slowing may be detectable in gut microbial communities before a state shift.

In all cases, we detected increasing lag‐1 autocorrelation with posterior evidence exceeding the level of statistical certainty (see Table 2).

**TABLE 2:** Comparison of EWS detection performance in publicly available case studies with different autoregressive models

Data	Model	*τ*	*p*‐Value
Climate	AR(1)	0.616	.126
	TVAR(1)	0.698	.018
	pTVAR(1)	0.492	<.001
Cyanobacteria	AR(1)	0.616	.118
	TVAR(1)	0.698	.014
	pTVAR(1)	0.332	.071
Gut microbiome (*Akkermansia*)	AR(1)	0.947	<.001
	TVAR(1)	0.008	.895
	pTVAR(1)	0.823	<.001

Note

In all cases the proposed probabilistic *p*TVAR(1) model detects an EWS with statistical certainty below the selected level α=1. For the nonprobabilistic comparison models the results are mixed.

**FIGURE 5:**
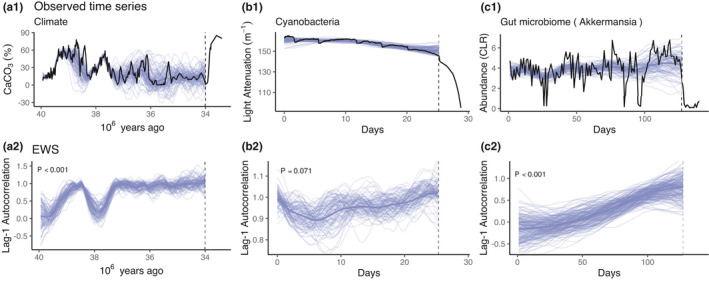
Application of the probabilistic EWS to real time series. (a1) The climate data depict sediment ${\text{CaCO}}_{3}$ levels which have been used as a proxy for the climate (Tripati et al., 2005). (b1) Experimental cyanobacteria data. Sudden relocations to lower abundance levels are due to experimental perturbations. (c1) An example of a gut microbiome time series that displays a state shift. CLR transformed abundance levels of the genus *Akkermansia* collapse at *T* = 128. The lag‐1 autocorrelation posteriors in the corresponding lower panels (a2–c2) provide evidence for rising trend at levels *p* < .001, *p* = .063 and *p* < .001 for the climate, cyanobacteria, and gut microbiome time series, respectively. In all figures, the black line displays the data and purple thin lines samples from a posterior, while the thicker line represents the posterior mean. Only the time series data preceding the observed state shift (dashed vertical line) was used in the analysis

In Figure 5, we present the fits of the proposed pTVAR(1) model. The model detects an EWS with statistical certainty in every data set and the time series trends are recovered with fidelity.

## 4 DISCUSSION

Complex ecological and other systems can undergo an abrupt and long‐lasting reorganization, a regime shift. Although such changes often arise unexpectedly, specific changes in the system oscillations can frequently be observed prior to such state shifts, providing early warnings of an anticipated transition. Despite the recent advances in developing early warning signals, practical challenges remain.

One of the key challenges for pragmatic application is the fine‐tuning and parameterizing the models for new data sets. Model parameterization can influence model performance in a data set‐specific manner (Dakos, Carpenter, et al., 2012) and only limited prior information is typically available to guide the choice of the ideal parameter ranges. Hence, the models may not be able to extract the full information from the data, or even lead to inconclusive or incorrect inferences. New methods that provide tools to automate this process and utilize all information from the data in an optimal way would be valuable for practitioners.

We have proposed a novel probabilistic formulation that aims to fill this gap in the current methodology and examine its performance in the context of early warning signal detection. Benchmarking the new method with related alternative approaches highlights the generic trade‐offs between robustness and sensitivity, and points to specific advantages that can be obtained with the probabilistic framework, thus informing the choice of the appropriate method in different application tasks. Our analysis focuses on autoregressive models, which have performed well in earlier studies (Dakos et al., 2008; Ives & Dakos., 2012; Veraart et al., 2012), and we have benchmarked three closely related statistical models that can be used to anticipate critical transitions in complex systems based on the analysis of time series autocorrelation structure. In the simulation study, we used an often used ecological model as the generative process with relatively low sample sizes of some dozens (range 17–189) as opposed to several hundreds in previous EWS studies (Carpenter & Brock, 2011; Dakos, Carpenter, et al., 2012; Dakos, Carpenter, et al., 2012).

The results from the probabilistic method are consistent with the available alternatives in real experimental data, while showing increased robustness to uncertainties regarding the data generating model structure and parameters. This is instrumental in the common scenario where the underlying mechanisms of the complex system dynamics are unknown. Moreover, the probabilistic approach is extendable and as such provides a new family of methods for early warning signal detection that can be used to naturally incorporate variable modeling assumptions and prior knowledge in the model as these become available. The smoothing bandwidth estimation differs among the methods but it in general reflects the amount of total information used at each time point. By systematically investigating a range of parameters, we can assess the model robustness with respect to bandwidth variation. The probabilistic formulation benefits from a flexible time‐varying parameterization and smooth regularization obtained through Gaussian process priors. Whereas the probabilistic method employs an adaptive smoothing scheme, the alternatives must resort to more indirect techniques, such as choosing the TVAR(1) kernel regression bandwidth with leave‐one‐out cross‐validation, or using a collection of bandwidth values to obtain a distribution of estimate of trend significance for a warning signal. While employing a collection of models with different hyperparameters is possible with probabilistic methods as well, in general this approach may be problematic when incorporating poorly performing bandwidth ranges. However, as we show, the probabilistic variant has a comparatively good fit to the data at all levels of smoothing, whereas in our experiments, the nonprobabilistic models had the tendency to underfit at larger bandwidth values.

Although trend fitting is only a secondary target in EWS detection, discrepancies like this can hint at deeper problems in the correlation structure estimation. Indeed, at larger bandwidth values, the TVAR(1) model produced essentially monotonous trajectories with an increasingly negative association with the true autocorrelation. Distinguishing true from false alarms is another important aspect of EWS detection. The TVAR(1) method claimed highly certain false‐positive early warnings. Whereas both time‐varying models had similar true positive rates in our experiments than the AR(1) model and thus better detection performance, the probabilistic model had a lower rate of false negatives than its nonprobabilistic counterpart. Interestingly, the simpler AR(1) model also had a relatively low rate of false negatives, which could be partially attributed to its reduced flexibility that can increase robustness. When measuring the overall classification accuracy with F1 score, the probabilistic model achieved the best performance at most levels observation error. Together, these results point to the need to include the quantification and analysis of false alarms when evaluating the performance of early warning indicators, which some studies have missed while focusing on the detection of true positives. Moreover, this highlights the problem of choosing the model hyperparameters. In realistic scenarios, it is not known whether or not the studied system is approaching a tipping point, or alternatively, a lot of data may be available and critical slowing down occurs at a relatively small proportion of the time series. In such cases, any predetermined hyperparameter values can lead to false findings, and one needs to be careful not to let any bias affect the modeling choices and not to try to optimize the models to get a desired result.

We also demonstrated the application of the compared methods on three public data sets, including earlier studies that reported increasing autocorrelation for the climate (Dakos et al., 2008) and experimental cyanobacterial time series (Veraart et al., 2012), and an additional example from gut microbiome times series (*Akkermansia* genus) (David et al., 2014), where a slowing down in its abundance dynamics could be observed prior to a collapse. However, the collapse occurred simultaneously as an onset of diarrhea in the study subject due to food poisoning and the associations between this potential EWS and the effects of later food poisoning event remain unknown.

**FIGURE A1:**
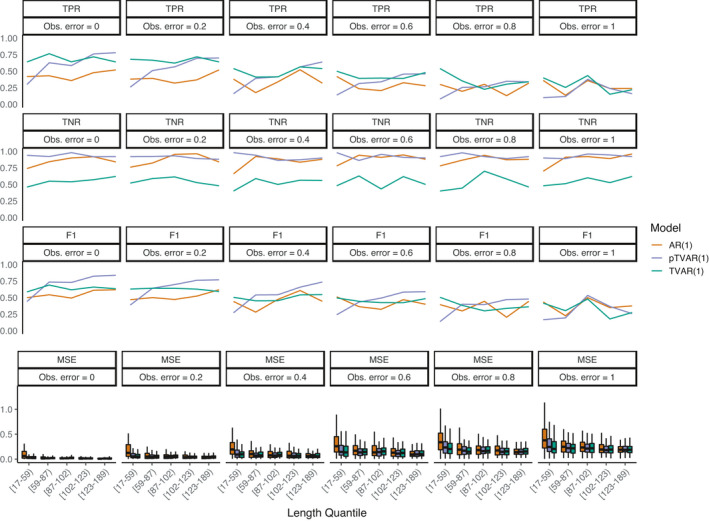
Comparison of EWS detection performance between different autoregressive models as a function of time series length. AR(1) is the standard autoregressive model, TVAR(1) the time‐varying autoregressive model, and *p*TVAR(1) the probabilistic time‐varying model. The panels depict results for different performance metrics and levels of observation error. TPR, TNR, F1, and MSE correspond to true‐positive rate, true‐negative rate, F1 score, and mean squared error between approximated and inferred autocorrelation trajectories, respectively

Our experiments show that the newly introduced probabilistic method can provide improvements in robustness over the alternatives. The probabilistic models could potentially provide also other advantages. First, probabilistic models provide a principled way of assessing statistical significance as the autocorrelation posterior distributions can be readily used to compute the evidence for a positive trend without resorting to surrogate data analysis, thus providing conceptual simplification and computational advantages compared to the alternatives. Second, specific knowledge of the system can be incorporated through probabilistic priors, and a comprehensive investigation of this option would deserve its own study. We employed the GP priors mainly to regularize the statistical learning in order to avoid overfitting. This emphasizes long‐term trends and reduces parameter sensitivity to large stochastic variations in the data. This comes at the cost of potentially missing real, rapidly occurring events but the continuity of the time‐varying process is a natural assumption in the context of EWS that often arise gradually under changing and constantly monitored conditions (Dakos & Bascompte, 2014). The EWS are typically concerned with more gradual trends as rapid changes are difficult to detect reliably in noisy and sparsely sampled data. In the simulation benchmarks, we used the length of the entire series as the length scale but also showed that this choice is not as sensitive as the bandwidth selection with the nonprobabilistic models. Real‐world data may, however, exhibit relatively long periods of constant conditions before conditions begin to change or strong nonstationary variations requiring customized length scales. We encountered this when analyzing the real data sets. An important potential advantage of incorporating prior information in the probabilistic models, for instance from previously collected time series of the same system, is that the prior can regularize the inference toward practically meaningful areas of the parameter space when only limited amount of data is available, potentially leading to more reliable and rapid inference. Hence, we expected the probabilistic pTVAR(1) model to outshine its nonprobabilistic comparison models on the shorter time series of the simulation benchmark. However, we found that the performance of pTVAR(1) decreased rapidly with very low sample sizes (Figure A1 in Appendix 1), which points to the need to study the role of the hyperparameters further. Third, the probabilistic modeling framework could be naturally extended to handle more complex modeling scenarios (Gelman et al., 2013). The probabilistic formulation could allow, for instance, aggregation of parallel observations with a hierarchical model, or modifying the observation or noise structure. For instance, a Poisson observation model could be used when modeling certain types of one‐dimensional count data. Explicit observation errors could be incorporated by separating the unobserved latent system state and the observation error process (Ives & Dakos, 2012), as in standard state space models that are typically fit with Kalman filters. These can, however, be challenging to implement and fit (Auger‐Méthé et al., 2016), and their added value remains currently unknown. Overall, the advances in the probabilistic formulation lead to conceptual simplifications in model interpretation and can provide agility.

The aim of this work is to present a novel probabilistic approach for EWS detection, but we leave comprehensive testing of the proposed model's properties for a later study. For instance, it would be valuable to test the model on noncritical transitions and in situations where increasingly sparse observations, or more observations farther away from the tipping point are available. Several other directions for further development can be envisioned. We have restricted our analysis to the TVAR(p) model with the lag p=1. Models with higher lags could provide a better fit to the data but such increasing model complexity could also lead to overfitting or problems in Bayesian posterior sampling, such as weak model identifiability when the sample sizes are limited (Luo et al., 2009). Another potentially fertile direction would to study the effect of different types of priors. Here, we used GP priors with the Matérn structure for the time‐varying parameters. While we experimented with other members of the Matérn kernel class, other alternatives might give better results. Ideally, domain knowledge about the system's dynamical properties should be used to guide such decisions (Gelman et al., 2013; Rasmussen & Williams, 2006). Prior distributions could also be used for the hyperparameters. By changing the length scale from a constant to a variable and giving it a prior, it would be possible to include sensitivity analysis in the fitting process and let the model give more weight to the "better" hyperparameter values. In our preliminary experiments, this approach encountered convergence issues in the MCMC algorithm, implying a need for further theoretical investigations. In the simulation study, we set the length scale to length of the time series. While we show that this is a good choice in most situations, we acknowledge that the selection process could be improved. Cross‐validation and other model selection techniques could be used to enhance EWS detection accuracy and automate the process further. A limitation in many time series models, including the standard autoregressive processes, is that they assume equidistant observations. Imputing or ignoring missing values in unevenly collected time points may be necessary in such cases, with potential information loss and distortions. The Bayesian framework would also allow a natural framework for incorporating missing data as random variables. Another potential solution would be to use time‐varying Gaussian processes as the generative process for the state variable (Heinonen et al., 2016). The Ornstein–Uhlenbeck process (an example of a GP) is the extension of the standard AR(1) process to the continuous domain. By adding time dependence to the covariance structure, it would be possible to generalize the model presented in this paper another step further. In our preliminary experiments, these models encountered convergence issues in the MCMC sampling and exhibited substantially higher computational costs. In general, a key drawback of using Gaussian processes is that the computation time scales in $O\left(n^{3}\right)$ meaning that analysis of longer time series can quickly become prohibitive.

Anticipating critical transitions in complex systems is a notoriously challenging and a largely unresolved task despite the recent progress in this area. Our current work focused on detecting changes in autocorrelation structure, a widely used and robust univariate indicator (Clements et al., 2015; Dakos, van Nes, et al., 2012). However, related probabilistic extensions could be implemented also in the context of other commonly reported EWS, such as variance or change point detection in the context of flickering (Dakos, Carpenter, et al., 2012). Extending some other established univariate EWS indicators (e.g., skewness, kurtosis, and spectral properties) to the probabilistic framework might be less straightforward in practice, however, since formulation of the equivalent generative models could be less obvious than for the autoregressive case. The methodological advances that we have proposed and investigated are generic, naturally extendable, and, as our experiments demonstrate, potentially applicable across a broad variety of application domains.

## CONFLICT OF INTEREST

All authors declared no conflict of interest.

## AUTHOR CONTRIBUTIONS

**Ville Laitinen:** Conceptualization (equal); Formal analysis (equal); Investigation (equal); Methodology (equal); Project administration (equal); Software (lead); Visualization (equal); Writing‐original draft (equal). **Vasilis Dakos:** Conceptualization (supporting); Methodology (supporting); Visualization (equal); Writing‐original draft (supporting). **Leo Lahti:** Conceptualization (equal); Formal analysis (equal); Funding acquisition (lead); Investigation (equal); Methodology (equal); Software (supporting); Supervision (lead); Visualization (equal); Writing‐original draft (equal).

## Data Availability

The data sets are available in the original sources. The source code for the experiments and simulated data generation is available at https://zenodo.org/record/4638525 .

## APPENDIX 1

### Fitting information

We implemented the Bayesian models in Stan (Stan Development Team, 2020) and sampled the posterior with the No‐U‐Turn Sampler (NUTS) variant of the Hamiltonian Monte Carlo algorithm.

The Gelman–Rubin statistic $\hat{R}$ remained below the standard 1.1 threshold (Gelman & Rubin, 1992), indicating model convergence. We encountered no divergent transitions in simulations. For each simulation, we used 2 chains with 2000 iterations and random initializations. We discarded the first half of samples as warm‐up and set the maximum tree‐depth and step size parameters to 15 and 0.95, respectively. The AR(1) model was fit with ordinary least squares regression as implemented in the R function *stats::ar.ols* and the TVAR(1) model using the function *tvReg::tvAR*.

We used the latent variable formulation of Gaussian processes to achieve more efficient posterior sampling (Kuss & Rasmussen, 2005). This formulation models the process as a latent one‐dimensional vector η that is mapped to the output space as θ=Lη, where L is the lower triangular matrix with positive diagonal from the Cholesky decomposition $k_{3/2}=LL$. Here, θ corresponds to the parameters $\phi_{t}$ and $\mu_{t}$. This parameterization reduces the dimensionality of the posterior distribution and this way speeds up the Markov chain Monte Carlo sampling process.
